# Hyponatraemia monitoring in those prescribed antidepressants - an audit from an inpatient older adult ward

**DOI:** 10.1192/bjo.2021.228

**Published:** 2021-06-18

**Authors:** Lorena Chavez, Jake Scott, Richard Hoile, Jane McNulty, Louisa Marchant-Rutherford

**Affiliations:** Sussex Partnership NHS Foundation Trust

## Abstract

**Aims:**

To assess follow-up of sodium levels for in-patients prescribed antidepressants in practice compare to the standard of 3 monthly sodium levels for all patients who are prescribed antidepressants and at risk of hyponatraemia

**Method:**

A list of the 20 most recently discharged patients from Meridian Ward, an older-adult functional inpatient ward, was prepared by the team administrator on 6th May 2020.

We audited the entire duration of our patient's stay on Meridian Ward (we did not include periods of their admission when they were on other wards) using the electronic notes system, Carenotes.

We also checked the electronic biochemistry results system, ICE, for sodium results, and the discharge summary for mentions of fluid restriction, medications and handover to GP of sodium-checking. We also checked scanned drug charts to see if they were on antidepressants and other implicated drugs.

For people with episodes of hyponatraemia, in order to retrieve further info we looked at discharge summary and searched the activity notes for the following terms

“Hyponat”

“sodium”

“fluid restrict”

“Low na”

We regarded the following conditions as risk factors for hyponatraemia:

cardiac

malignancy

respiratory

hypothyroid

renal

hepatic

stroke

We regarded following medications as risk factors:

opioids

diuretics

carbamazepine

theophylline

antipsychotics

NSAIDs

PPIs

ACE-I

ARBs

amiodarone

domperidone

sulphonylureas

**Result:**

14 of the 20 patients were taking antidepressants. Of those: 13 were eligible for regular sodium monitoring due to risk factors 11 of these had 3-monthly sodium levels during admission For only 2 of these did we make a plan for the GP to continue to monitor the sodium level in community 3 had an episode of hyponatraemia implicated antidepressants: sertraline plus mirtazapine mirtazapine (very serious episode which caused seizure) sertraline for 2 of them an appropriate plan was made 1 without a plan - a mild hyponatraemia with nothing documented in the notes

**Conclusion:**

During their admission to Meridian Ward, 85% of patients taking antidepressants who had risk factors for hyponatraemia had three-monthly sodium levels in line with the trust guidance. However, only two patients (15%) had a plan for further sodium levels in the discharge summary sent to the GP. This highlights a need for improved awareness of risk factors for hyponatraemia and, in particular, improved communication with general practitioners who are going to take over prescribing of antidepressant medications.

Recommendations

3 monthly Na levels for all patients with risk factors

i.e. on any antidepressant prescribed PLUS any one of:

>80 years

History of low sodium

AKI during admission

Relevant comorbidities (see above)

>1 antidepressant

Other meds that can cause hyponatraemia

More frequent monitoring for all those with with multiple risk factors AND who are starting/increasing antidepressant:

baseline sodium plus repeat after 2 and 4 weeks

Communicate to GP the need for 3-monthly sodium monitoring for those with above risk factors

Re-audit in 6-12 months’ time

